# Type VI Aplasia Cutis Congenita: Bart's Syndrome

**DOI:** 10.1155/2015/549825

**Published:** 2015-11-01

**Authors:** Ferit Kulalı, Ahmet Yagmur Bas, Yusuf Kale, Istemi Han Celik, Nihal Demirel, Sema Apaydın

**Affiliations:** ^1^Division of Neonatology, Etlik Zübeyde Hanim Women's Health Teaching and Research Hospital, Ankara, Turkey; ^2^Department of Pathology, Dr. Sami Ulus Maternity and Children Research and Training Hospital, Ankara, Turkey

## Abstract

Bart's syndrome is characterized by aplasia cutis congenita and epidermolysis bullosa. We present the case of a newborn male who developed blisters on the mucous membranes and the skin following congenital localized absence of skin. Bart's syndrome (BS) is diagnosed clinically based on the disorder's unique signs and symptoms but histologic evaluation of the skin can help to confirm the final diagnosis. The patient was managed conservatively with topical antibacterial ointment and wet gauze dressing. Periodic follow-up examinations showed complete healing. We emphasized that it is important to use relatively simple methods for optimal healing without the need for complex surgical interventions.

## 1. Introduction

Bart's syndrome (BS) is characterized by aplasia cutis congenita (ACC) and epidermolysis bullosa (EB). It may be accompanied by nail abnormalities such as congenital absence, nail dystrophy, or further loss [1]. Its etiology and pathophysiology are still controversial although several hypotheses have been proposed [2].

Although the inheritance pattern appears to be autosomally dominant, isolated cases have been recognized. Lesions in BS are usually unilateral and involved the medial and/or dorsal surface of the limbs. They appear on extremities as sharply demarcated, glistening red ulceration that extend upward from the dorsal and the medial surface of the foot to the shin [3]. In this paper, the management of a sporadic case of Bart's syndrome is presented.

## 2. Case

A male infant was born to a 25-year-old gravida 2, para 2, mother via cesarean section at term. Labor and delivery were uncomplicated. Apgar scores were 8 and 9 at 1 and 5 minutes, respectively. The parents were not relatives, and the family history was unremarkable. They had a healthy, five-year-old girl. In their family there was no history of skin, connective tissue, or autoimmune disease.

**Figure 1:**
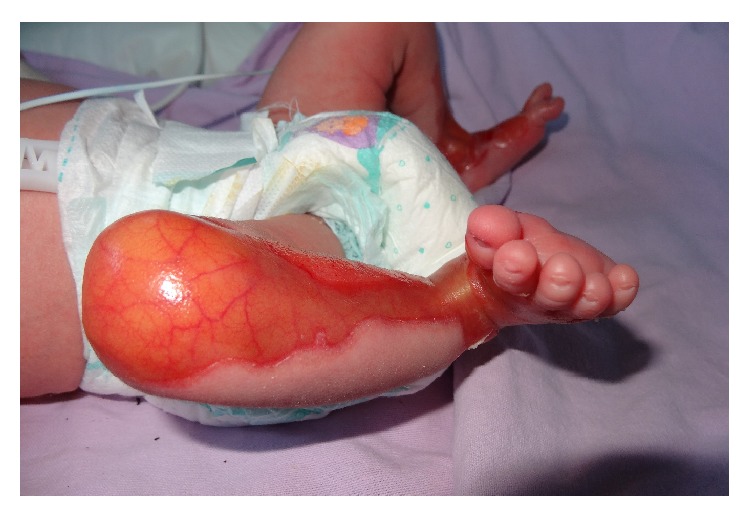
Sharply demarcated lesion margin.

**Figure 2:**
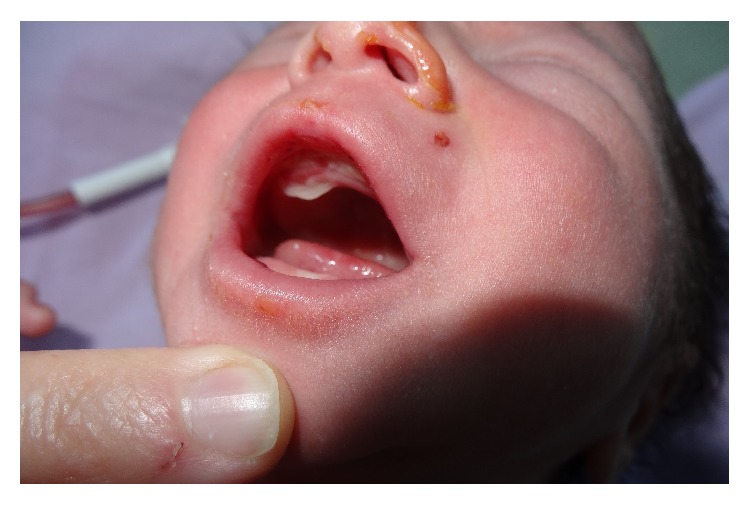
Blisters on the upper lip mucous membranes.

**Figure 3:**
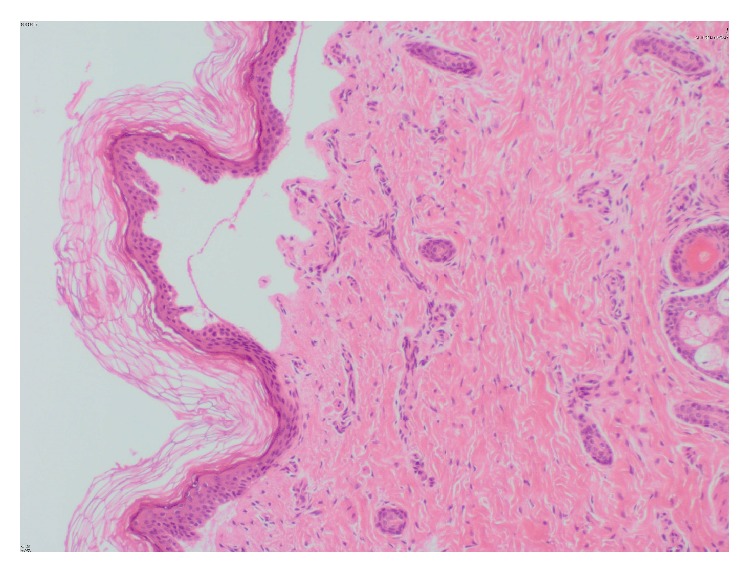
Subepithelial vesicle formation.

He had normal weight, length, head circumference, and vital signs. On physical examination, he was found to have absence of skin over the anteromedial aspect of both lower legs, starting from the knees and extending to dorsal and medial plantar aspect of the feet. The lesions have sharply demarcated borders covered by a red ultrathin translucent membrane (Figure 1). On the second day, he developed blisters on the upper lip mucous membranes and on the left wrist (Figure 2). Results of his complete blood count, liver and renal function tests, electrolytes, ionized calcium, and magnesium were within normal limits. Serologic tests for TORCH were negative. Ophthalmological examination, abdominal and cranial ultrasound screening, and echocardiography revealed normal findings. Histopathological examination of the left wrist lesion demonstrated full or partial developed subepithelial vesicle formation (Figure 3). Direct immunofluorescent staining showed weak focal perivascular deposition of IgM, IgA, C3, and Fibrinogen but did not demonstrate IgG, C4, and C1q depositions that have suggested dystrophic epidermolysis bullosa. The combination of aplasia cutis congenital (ACC) and EB led to the diagnosis of Bart's Syndrome.

Patient was referred to dermatology. Physician recommended to allow the area to heal spontaneously by using conservative wound care such as topical antibacterial ointment and wet gauze dressing two to three times a day. The patient was treated by applying topical mupirocin (Bactroban) and wet gauze dressing (Bactigras) for 4 weeks and skin lesions had recovered substantially with appropriate therapy. The infant was discharged on the tenth day of hospital stay to continue local wound care by his mother.

## 3. Discussion

Bart's syndrome was originally described in a family with congenital absence of skin on the lower leg and widespread blistering of skin, mucous membrane, and nail dystrophy [3]. Previously, Frieden (1986) classified by location and presence of the other anomalies into nine groups. According to this classification, group 6 was defined as Bart's syndrome which is characterized by a combination of congenital localized absence of skin (CLAS) and EB [4]. Currently, however, we know that Bart's syndrome is characterized by EB with congenital absence of skin and is one of the subtypes of EB [5]. BS is sometimes accompanied by nail abnormalities which are not absolutely required for making the diagnosis. In our patient, there was involvement of congenital localized absence of skin and blistering lesions but there was no involvement of nail.

The scalp is the most frequent site of involvement, although trunk and extremities may also be involved [6]. ACC may be unilateral or less frequently bilateral. Our patient's lesions had symmetric involvement at both legs. BS can also be associated with other anomalies as pyloric atresia, rudimentary ear development, flattened nose, broad nasal root, and wide-set eyes [7]. The present case had no associated anomalies.

BS is diagnosed clinically based on the disorder's unique signs and symptoms but histologic evaluation of the skin can help to confirm the final diagnosis [2]. Biopsy specimen revealed an increased inflammatory infiltrate in the dermis, probably because of capillary leakage.

Zelickson et al. demonstrated various abnormalities of anchoring fibrils, which are mainly composed of type VII collagen, at the dermal-epidermal junction in BS [8]. Duran-McKinster et al. proposed that congenital localized absence of skin in BS may follow the lines of Blaschko owing to physical trauma in utero [1].

Christiano et al. identified a mutation leading to a glycine-to-arginine substitution at type VII collagen [9]. The genetic abnormality has been associated with chromosome 3, with an autosomal pattern of inheritance. Molecular (DNA) analysis for the diagnosis could not be performed in the present case.

The predominant inheritance pattern is autosomally dominant, while some cases with unaffected parents are believed to occur due to sporadic mutation [10]. As shown in the study by Chiaverini et al. sporadic cases have been reported to be associated with mutations of the triple helix domain of collagen VII gene. This mutation that is correlated with synthesis of a thermolabile Col 7 may lead to BS. The present case had no family history of congenital localized absence of skin and blistering lesions; therefore he was a sporadic one. The different diagnosis of BS includes aplasia cutis congenita, epidermolysis bullosa, Adams-Oliver syndrome, and congenital bullous poikiloderma (Kindler syndrome).

The management of BS is usually conservative, preventing infection of affected area and allowing the affected portion to declare itself in order to optimize future reconstruction. The goal of treatment is to accelerate healing and reduce the risk of scarring [11]. Close follow-up for serious complications, such as hemorrhage, infection, hypothermia, and hypoglycemia, is important. Prognosis is good and depends on efficacy of treatment.

**Figure 4:**
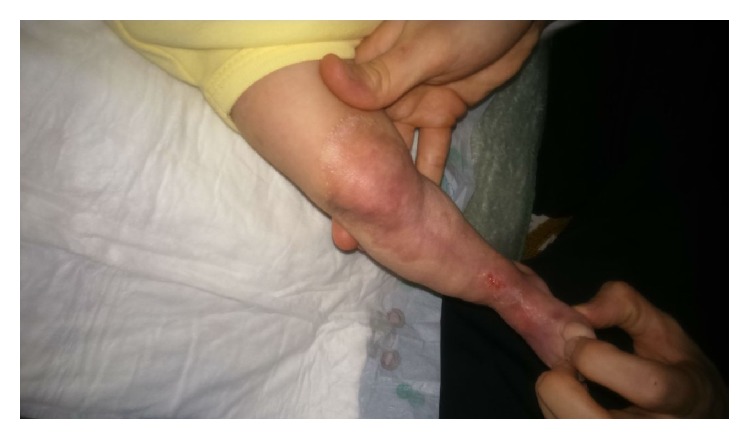
Healing of the lesions with conservative treatment.

The use of prophylactic systemic antibiotics in therapy has not been recommended. We used topical antibacterial ointment and wet gauze dressing in our patient. Sterile dressings with Bactigras of the lesions were done twice daily. Epithelialization process on the lesions was noted on the tenth day of hospital stay and the infant was discharged. As shown in Figure 4, the wound healing in this patient is rapid, efficient, and perfect.

We emphasized that it is important to use relatively simple methods for optimal healing without the need for complex surgical interventions.
